# Editorial: Plant-based solutions for sustainable agriculture and environmental remediation

**DOI:** 10.3389/fpls.2025.1729054

**Published:** 2025-11-20

**Authors:** Carla S. Santos, Mehrdad Zarafshar, Marta Nunes da Silva

**Affiliations:** 1 Universidade Católica Portuguesa, CBQF – Centro de Biotecnologia e Química Fina – Laboratório Associado, Escola Superior de Biotecnologia, Porto, Portugal; 2 Department of Forestry and Wood Technology, Faculty of Technology, Linnaeus University, Växjö, Sweden

**Keywords:** nutrient cycling, mulching, grassland degradation, phytoremediation, deep learning - artificial intelligence, phytochemical profile, glyphosate, weed management

The escalating global challenges of climate change, environmental degradation, and food insecurity call for urgent, transformative approaches to agriculture and ecosystem management. With the world population projected to exceed 9 billion by 2050, pressure on finite natural resources continues to intensify, exacerbating soil degradation, biodiversity loss, and pollution (Cobourn and Ignaciuk, 2025). To sustain long-term productivity and ecological function in forest plantations, Fan et al. emphasize the importance of age-specific nutrient management. Their findings reveal a shift from nitrogen limitation and high phosphorus resorption efficiency in young stands to phosphorus limitation and elevated nitrogen resorption efficiency in older stands (>100 years). Stable and high leaf C:N:P ratios indicate low nutrient use efficiency, while seed nutrient composition in mature stands, characterized by higher crude fat and protein, highlights the need for long-term strategies that prioritize nutrient cycling efficiency. In arid orchard systems, Li et al. demonstrate that mulching practices, particularly plastic film (FM) and straw mulching (SM), significantly enhance pear yield and fruit quality. Field treatments resulted in significantly higher fruit yield and quality traits, such as sugar/acid ratio and organic acid composition. Mulching further enhanced soil nutrient availability (including NH_4_^+^-N, K, Ca, Fe, Mn, Cu, and Zn) and enriched rhizosphere microbial communities, including beneficial bacteria like Proteobacteria, Acidobacteria, Gemmatimonadetes, and Firmicutes, underscoring its role in promoting soil health and agroecosystem resilience.

However, assessing soil quality and ecosystem health remains challenging due to complex interactions among biophysical, socio-economic, and technological factors operating across scales (Bond et al., 2024). To address this, Zhang et al. introduce a geo-coding and abrupt analysis-based (GAAB) method to diagnose grassland degradation via the Living Status of Vegetation (LSV), a composite index integrating cover, height, biomass, and species diversity. Applied to subalpine meadows, GAAB revealed uneven degradation thresholds and asynchronous shifts in community structure and function, including a species transition from *Dactylis glomerata* in healthy meadows to *Potentilla lineata* in degraded zones, potentially enabling tailored restoration strategies based on degradation severity.

Beyond their role as soil health indicators, plants are pivotal agents in soil restoration due to their adaptive capacity and biochemical versatility. For example, phytoremediation, using plants to extract, degrade, or immobilize pollutants, has shown efficacy in mitigating contaminants across soil and aquatic systems (Ranđelović et al., 2025; Sahoo et al., 2025). Advances in artificial intelligence, including deep learning and process modelling, can further enhance plant-based monitoring of environmental health, ecological restoration, and sustainable agriculture (Yeasmin et al., 2024; Wu et al., 2024; Dong et al., 2024). In this context, Guo et al. introduce Bio-DANN, an integrated framework that couples process based biogeochemical simulation with deep learning for dynamic pollutant monitoring and ecological restoration in agricultural waste systems. Bio-DANN mechanistically simulates plant mediated absorption, transformation, and degradation of contaminants, while ingesting environmental sensor streams (e.g., soil moisture and temperature) to train attention-based neural networks. By fusing phytoremediation processes with data driven inference, Bio-DANN delivers accurate predictions of pollutant concentrations and restoration trajectories, outperforming prior approaches in real time monitoring and multidimensional ecological assessment.

Concurrently, Nazari et al. highlight the value of integrating molecular and environmental data to guide sustainable cultivation and conservation. ITS-based phylogenetic analysis of Iranian Papaver species revealed complex evolutionary relationships and introgression, while phytochemical profiles, dominated by phenolics, flavonoids, and alkaloids, correlated more strongly with environmental variables (e.g., humidity, elevation) than genetic distance. Among the genotypes evaluated, *P. hybridum* exhibited the highest antioxidant activity, while *P. macrostomum* and *P. dubium* showed higher flavonoid and anthocyanin levels, underscoring their pharmaceutical potential. Valorizing locally adapted underutilized crops and crop wild relatives has proven effective in sustaining key ecosystem services and promoting more sustainable agrifood chains, in part because these species often require fewer chemical inputs (Silva et al., 2025). Their inclusion in diversified cropping systems can therefore help reduce pesticide dependency, a global concern associated with biodiversity loss, declining water quality, harm to non-target organisms, and the spread of resistant pest populations. Beyond pests, weeds remain a major challenge because they compete with crops for nutrients, water, and light, frequently driving additional fertilizer use to offset yield losses. Addressing weed competition, Li et al. investigated targeted mutations in the EPSPS gene, which encodes a key enzyme of the shikimate pathway, to enhance glyphosate resistance in *Nicotiana tabacum*, allowing for lower application frequency and dosage, and potentially reducing environmental contamination and herbicide runoff. In addition, transgenic lines showed improved oxidative stress resilience, which could support plant survival in degraded or polluted soils, thus contributing to agroecosystem stability.

Collectively, this Research Topic illustrates how plant-based solutions can deliver measurable gains in productivity, resilience, and environmental quality, particularly when deployed as integrated systems rather than isolated interventions. From nutrient cycling in ancient forest stands to microbial modulation in arid orchards, from phytochemical diversity in underutilized species to AI-enhanced pollutant monitoring, the evidence is clear: plant-based solutions thrive when embedded in systems thinking, supported by robust data, and aligned with ecological principles. Effective implementation will depend on participatory codesign of resilient landscapes with farmers, foresters, and local communities, in which biodiversity is valued, external inputs reduced, and ecosystem services quantified and incentivized. As such, the path forward demands not only innovation but integration, where transdisciplinary research converges to embed evidence-based insights into holistic, system-oriented decision-making frameworks.
